# Natural products from traditional medicine as promising agents targeting at different stages of oral biofilm development

**DOI:** 10.3389/fmicb.2022.955459

**Published:** 2022-08-12

**Authors:** Yaqi Chi, Ye Wang, Mengzhen Ji, Yanyao Li, Hualing Zhu, Yujia Yan, Di Fu, Ling Zou, Biao Ren

**Affiliations:** ^1^State Key Laboratory of Oral Diseases, National Clinical Research Center for Oral Diseases, West China Hospital of Stomatology, Sichuan University, Chengdu, China; ^2^State Key Laboratory of Oral Diseases, National Clinical Research Center for Oral Diseases, Department of Endodontics, West China Hospital of Stomatology, Sichuan University, Chengdu, China

**Keywords:** natural products, antibiofilm effect, infection, microbiome balance, biofilm formation

## Abstract

Oral cavity is an ideal habitat for more than 1,000 species of microorganisms. The diverse oral microbes form biofilms over the hard and soft tissues in the oral cavity, affecting the oral ecological balance and the development of oral diseases, such as caries, apical periodontitis, and periodontitis. Currently, antibiotics are the primary agents against infectious diseases; however, the emergence of drug resistance and the disruption of oral microecology have challenged their applications. The discovery of new antibiotic-independent agents is a promising strategy against biofilm-induced infections. Natural products from traditional medicine have shown potential antibiofilm activities in the oral cavity with high safety, cost-effectiveness, and minimal adverse drug reactions. Aiming to highlight the importance and functions of natural products from traditional medicine against oral biofilms, here we summarized and discussed the antibiofilm effects of natural products targeting at different stages of the biofilm formation process, including adhesion, proliferation, maturation, and dispersion, and their effects on multi-species biofilms. The perspective of antibiofilm agents for oral infectious diseases to restore the balance of oral microecology is also discussed.

## Introduction

The oral cavity represents a favorable habitat for over 1,000 species of microorganisms, including viruses, bacteria, archaea, and fungi, due to its moist condition and suitable temperature (Marsh et al., 2011; Morse et al., 2018). Most oral microorganisms exist in the form of biofilms (Hu et al., 2019). Maintaining the ecological balance between the human host and intrinsic oral microorganisms is essential for oral health (Lof et al., 2017; Bacali et al., 2022). However, the dysbiosis of oral microbiota may promote the growth of some pathogenic species to form the oral pathogenic biofilms, which cause many oral infectious diseases such as caries, apical periodontitis, and periodontitis (Lof et al., 2017). These diseases have highly increased economic pressure and seriously affected global public health (2020) (Olusanya et al., 2020).

In recent years, the overuse of antibiotics in infectious diseases has gradually challenged their clinical treatment due to the rapid increase in drug resistance (Tent et al., 2019). Moreover, the broad-spectrum antimicrobial effects of antibiotics have been proved to cause the microecological dysbiosis (Kuang et al., 2018). Therefore, many non-traditional treatments have been developed, such as the application of virulence disruptors, immunomodulators, phage therapies and so on (Tse et al., 2017). The natural products from traditional medicine have been proved to be one of the practical alterative for antibiotics in infectious diseases (Melander et al., 2020). The various functions, high safety and low cost of natural products also highlight their future broader application in clinical practice (Fan et al., 2021).

Oral infectious diseases are mainly caused by biofilms (Marsh and Zaura, 2017). To better understand the mechanisms and functions of natural products from traditional medicine against oral biofilms, we summarized and discussed the antibiofilm effects and mechanisms of natural products targeting at the different stages of oral biofilm formation. We also highlighted that restoring the balance of oral microecology without killing oral microorganisms broadly was a preferable way to develop new antimicrobial agents for oral infectious diseases.

## Oral microbiome and its importance in oral cavity

According to the Human oral microbiome database (HOMD), there are nearly 150 genera and 700 prokaryote species in human oral cavity, and 96% of which are classified in six phyla (Gao et al., 2018). Over 250 species have been cultured and characterized; however, 20–60% of the species in the oral microbiome are unculturable currently. Some of the oral bacteria have been confirmed as the main pathogens of oral infectious diseases (Sedghi et al., 2021). For example, *Streptococcus mutans* and *Porphyromonas gingivalis* are considered as the key pathogens to cause caries and periodontitis, respectively. Beyond the bacterome, oral mycobiome is relatively rare (<0.1% in microbiome) and has not been well characterized (Baker et al., 2017). In recent years, large evidence proved the interaction between oral infectious diseases and oral microbiome, such as caries (Bowen et al., 2018), periodontitis (Lamont et al., 2018), and oral cancer (Irfan et al., 2020). Many systematic diseases, such as those from the gastrointestinal system and nervous system are also associated with oral microbial dysbiosis (Peng et al., 2022). The relationships between oral microbiome and oral or systematic health highlight the importance to maintain the balance of oral microbiome.

## Mechanism of biofilm formation and its significance

**Figure 1:**
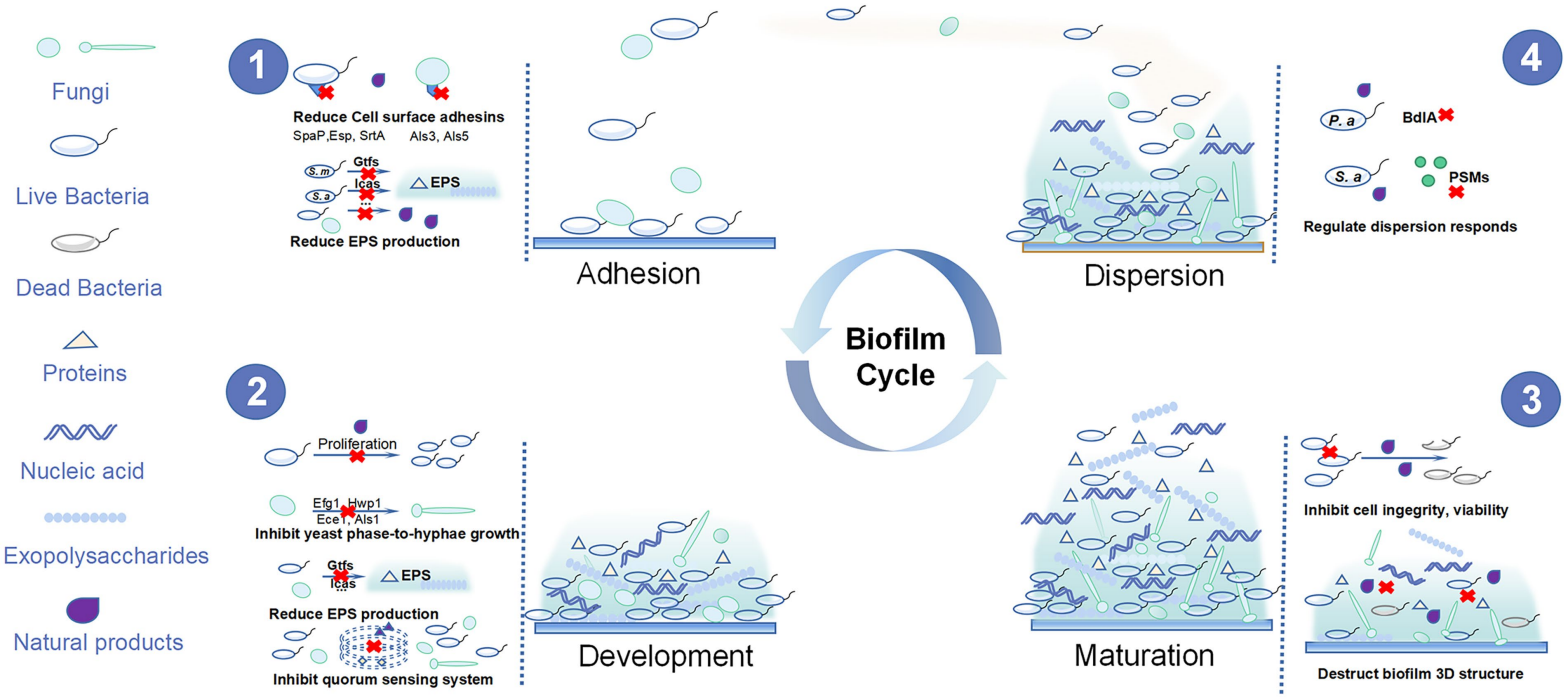
The four steps of oral biofilm formation and the anti-oral biofilm targets of natural products.

Biofilms are composed of microbial cells living in a dynamic and structured manner as well as the three-dimensional (3D) extracellular matrix of polymeric substances such as exopolysaccharides, proteins, and nucleic acids (Klein et al., 2015). Oral biofilm development can be roughly divided into four steps: adhesion, proliferation, maturation, and dispersion (Figure 1). Initially, the planktonic cells reversibly adhere to the tooth surface or other niches in oral cavity. Then, they interact with polymeric substances to form an irreversible adhesion and build three-dimensional structures. Finally, the matured biofilm displays motility characteristics and disperses to other surfaces, starting the same cycle (Stoodley et al., 2002; Mann and Wozniak, 2012). Biofilms have higher virulence and reduced susceptibility to antimicrobial agents compared with planktonic microorganisms due to the following aspects: first, the abundance of extracellular polysaccharides in biofilms can surround the microbial cells, thus restricting the penetration of antibacterial agents (Ciofu et al., 2017); second, the gradient of the micronutrient composition of the biofilms allows opportunistic pathogens to survive in nutrient-limited areas where they may become dormant and resistant to antibiotics (Ciofu et al., 2017); third, reciprocal, symbiotic, and antagonistic relationships occur among different species in the biofilm community. Their interactions can be affected by the environment, nutrition, and other factors to promote the resistance of biofilms to antibacterial agents. Moreover, the quorum sensing (QS) systems activated in the biofilm also increase the virulence and survival rate of biofilm microorganisms (Marsh et al., 2011; Hu et al., 2019). Thus, biofilm infection is more difficult to control and it is necessary to find new approaches for the treatment and prevention of biofilm infection.

## Advantages of antibiofilm therapy

Antibiofilm therapies are highly effective in controlling oral biofilm infections. For example, the usage of tetracycline, doxycycline, and minocycline in the clinical treatment of periodontitis resulted in a great effect on the primary outcome (probing pocket depth, PPD; Matesanz-Pérez et al., 2013). Except the antibiotics, many other therapies, including surgical therapy, bacteriophages and natural products, are also available to control oral biofilm infections with different specific advantages (Tse et al., 2017). Natural products, especially those from traditional medicine, are available from wide sources with various biological activities, which are well practical resources for oral biofilm control (Melander et al., 2020). Natural products have already been applicated in clinical practice for hundreds of years, thus, they are more cost-effective and safe in the controls of biofilm infections (Atanasov et al., 2021).

## Antibiofilm effects and mechanisms of natural products targeting at the different stages of biofilm formation

Natural products have become a hot research topic in recent years due to their promising antibacterial effects and various mechanisms. Many natural products have shown effective potential in inhibiting oral biofilms and controlling biofilm-related diseases, such as caries and periodontitis (Zheng et al., 2011). However, the pharmacological mechanisms of most natural products are not fully elucidated, and their mechanisms of inhibiting biofilm formation may be quite different from those of inhibiting the planktonic microorganisms. Biofilm development can be roughly divided into four stages: adhesion, proliferation, maturation, and dispersion. Here, we summarized and discussed the natural products targeting at the different stages of oral biofilm development (Table 1).

**Table 1:** Anti-oral biofilm natural products and their molecular mechanisms.

Plant extracts/ compounds	Mechanism	Target bacteria	Antibiofilm effect	Reference
Propolis	Inhibiting SpaP and glycosyltransferases enzymes (GtfB, GtfC, GtfD)	*S. mutans*	Decreased adhesion rate and EPS production.	Veloz et al. (2016)
Curcumin	Inhibition of SrtA, Gbps, Gtfs, Ftfs gene expression.	*S. mutans*	Decreased biofilm viability: 84.059%. Decreased biofilm thickness and EPS production.	Li et al. (2020b)
Curcumin	Inhibition of SpaP, Gtfs, SrtA, ComCD, and LuxS gene expression.	*S. mutans*	Decreased EPS production and biofilm formation.	Li et al. (2018)
Curcumin	Inhibition of key adhesins (Als1 and Als3) gene expression, promotion of genes related to aggregation (Als5 and Aaf1).	*C. albicans*	Decreased biofilm formation, initial adhesion, and promotion of *Candida albicans* aggregation.	Alalwan et al. (2017)
Theaflavins	Inhibition of Gbps and Gtfs	*S. mutans*	Decreased virulence factors (adherence, acid production, and EPS production) and biofilm formation.	Kong et al. (2021)
Sodium new houttuyfonate	Inhibition of Gtfs, quorum sensing	*S. mutans*	Decreased biofilm formation, EPS production, and quorum sensing. (100 μg/ml).	Shui et al. (2021)
Sodium houttuyfonate	Inhibition of BdlA (biofilm dispersion regulator) and FliC (gene related to flagella-mediated swimming motility) gene expression and pyocyanin production.	*P. aeruginosa*	Decreased biofilm formation, virulence factors, and inhibition of biofilm dispersion.	Shao et al. (2013); Wang et al. (2019)
Sodium New Houttuyfonate	Inhibition of Ras1-cAMP-Efg1 pathway related genes.	*C. albicans*	Decreased biofilm formation, adhesion, and change in the morphology of cells.	Wu et al. (2020)
EGCG	Inhibition of Gtfs, Ftfs gene expression.	*S. mutans*	Decreased biofilm viability:97% (4.4 mg/ml) and decreased EPS production.	Schneider-Rayman et al. (2021)
Green tea extract and EGCG	Inhibition of genes related to host colonization (FimA, HagA, HagB), tissue destruction (RgpA, Kgp), and heme acquisition (Hem).	*P. gingivalis*	Decreased biofilm initial adhesion and quorum sensing.	Fournier-Larente et al. (2016)
Tea extract/EGCG	Inhibition of H_2_S production.	*F. nucleatum*	Decreased biofilm formation, adhesion; inhibition of the growth and hemolysis and hydrogen sulfide production	Ben Lagha et al. (2017)
Water extract of *Galla chinensis*	Inhibition of IcaABD, YycFG gene expression and carbohydrate metabolic processes.	MRSA	Decreased biofilm formation and EPS production.	Wu et al. (2019)
Aloe-emodin	Inhibition of extracellular proteins and PIA production.	*S. aureus*	Decreased adherence, extracellular matrix production and biofilm formation.	Xiang et al. (2017)
Emodin	Inhibition of biofilm-related genes (DltB, SarA, SrtA, AgrA, IcaA, CidA).	*S. aureus*	Decreased biofilm formation and eDNA (importance to initial adherence) level.	Yan et al. (2017)
Baicalin	Inhibition of genes related to acid production (Idh), quorum sensing (ComX), and biofilm formation (FtsZ, GtfC, GbpB VicR, LuxS and BrpA)	*S. mutans*	Decreased acid production and biofilm formation.	Elango et al. (2021)
Baicalin	Inhibition of virulence-related gene expression and suppression of T3SS *via* PqsR of the PQS System	*P. aeruginosa*	Decreased virulence factors, especially T3SS.	Zhang et al. (2021a)
Berberine	Inhibition of SrtA and esp. gene expression.	*E. faecalis*	Decreased biofilm formation and promotion of biofilm dispersion.	Chen et al. (2016)
Berberine	Inhibition of the aggregation of PSMs into amyloid fibrils.	MRSA	Decreased biofilm formation and extracellular amyloid fibrils production.	Chu et al. (2016)
Allicin	Inhibition of Hwp1 gene expression.	*C. albicans*	Decreased biofilm formation.	Khodavandi et al. (2011)
Farnesol	Inhibition of the Ras1-Cdc35-PKA-Efg1 pathway	*C. albicans*	Decreased hypha formation.	Davis-Hanna et al. (2008)
Luteolin	Inhibition of Agr quorum sensing system	*S. aureus*	Decreased biofilm formation and initial adhesion.	Yuan et al. (2022)
Quercetin	Inhibition of quorum sensing system related gene expression (LasI, LasR, RhlI and RhlR)	*P. aeruginosa*	Decreased biofilm formation and virulence factors (pyocyanin, protease and elastase).	Ouyang et al. (2016)
Coumarin compound DCH	Competitively bind to the arginine repressor ArgR.	MRSA	Decreased biofilm formation.	Qu et al. (2020)
*Rhodiola rosea*	Inhibition of Gtfs gene expression and quorum sensing system.	*S. mutans*	Decreased biofilm formation and EPS production.	Zhang et al. (2020a)
Paeoniflorin	Inhibition of LuxS/AI-2 system.	*S. suis*	Decreased biofilm formation and EPS production.	Li et al. (2021)
*Macaranga tanarius*	Inhibition of hypha/biofilm-related genes (Ece1 and Hwp1) and reduction in cell aggregation.	*C. albicans*	Decreased biofilm formation.	Lee et al. (2019)

### Targeting at the cell colonization and adhesion

Adhesion is the initial step in biofilm formation and preventing the adhesion is the key strategy in controlling biofilm-related diseases. Oral microbial cells are reversibly attached to solid or non-solid surfaces and are further encapsulated by extracellular polymeric substances (EPS). The microcolonies formed by microorganisms and EPS are a hallmark feature of biofilms (Nadar et al., 2022). The interaction between microorganisms and substratum through specific protein receptors is essential during the adhesion process. Thus, the disruption of the interaction between microorganisms and substrate surfaces (such as cell surface-associated adhesins, EPS) can effectively prevent biofilm formation.

#### Effect of natural products on cell adhesion

Natural products have strong anti-adhesion effects on both bacteria and fungi. For some gram-positive oral bacteria, such as *Streptococcus* spp. and *Staphylococcus* spp., previous studies have shown that curcumin and tea extracts had excellent anti-adhesion effects. Curcumin, a natural product isolated from *Curcuma longa* (turmeric), decreased *Streptococcus mutans* adhesion to glass at a concentration of 8 μg/ml (Song et al., 2012) and inhibited 34–66% adhesion of *Staphylococcus aureus* to human keratinocytes (HaCaT) at 4.375 μmol/l (Sardi et al., 2017). Tea catechin epigallocatechin gallate (EGCG), a polyphenol extracted from tea, inhibited *S. mutans* adhesion in a dose-dependent manner at 7.8–31.25 μg/ml and reduced cell adhesion by 98.33% at 2 h (Xu et al., 2012). Similarly, the inhibition rate of emodin, an anthraquinone isolated from Chinese rhubarb, was 65% at the concentration of 4 μg/ml (Xiang et al., 2017). More importantly, natural products have also shown an excellent anti-adherent effect on antibiotic-resistant strains such as methicillin-resistant *Staphylococcus aureus* (MRSA). Curcumin showed an inhibitory effect at 8.65 μmol/l against MRSA (Sardi et al., 2017). For gram-negative oral species, EGCG showed a dose-dependent inhibition on *Porphyromonas gingivalis* adhesion at a concentration of 25–62.5 μg/ml (Fournier-Larente et al., 2016), whereas it inhibited *Fusobacterium nucleatum* adhesion at 125 μg/ml (Ben Lagha et al., 2017). Natural products can also inhibit the adhesion of oral fungi. Raspberry extracts (fruit of a shrub in Europe and northern Asia) exhibited a strong anti-adhesion effect on *Candida* spp. at 100 μg/ml (Dutreix et al., 2018), and curcumin prevented *Candida albicans* adhesion at 50 μg/ml (Alalwan et al., 2017).

#### Anti-adhesion mechanisms of natural products

##### Reduction in cell surface adhesins

SpaP (also known as antigen I/II, Pac, P1, and antigen B) represents a series of proteins that contribute to cell-surface adhesion and can be encoded by the SpaP genes in oral bacteria (Brady et al., 2010). Propolis is a resinous substance collected by bees. SpaP gene expression in *S. mutans* was reduced by nearly 80% after being treated with 0.1 μg/ml polyphenol-rich extract from propolis (PEP), which was even better than chlorhexidine (CHX), a commonly used cation antimicrobial agent in oral cavity (Veloz et al., 2016). Similarly, the expression of SpaP gene in *S. mutans* was downregulated approximately one-fold after being treated with curcumin, which was similar with CHX (Li et al., 2018). Baicalin is a hydroxy-flavone extracted from the genus *Scutellaria*. SpaP gene expression of *S. mutans* was downregulated after baicalin treatment, indicating the ability of baicalin to reduce oral bacterial cell adhesion (Elango et al., 2021).

Glucan binding proteins (Gbps) can mediate bacterial aggregation. GbpB is crucial in the initial sucrose-dependent biofilm formation and cell shape maintenance in *S. mutans* (Duque et al., 2011). Gbps is also an essential protein involved in cell-surface adhesion (Zhu et al., 2009). Theaflavins (TFs), a bioactive component of black tea (Zhang et al., 2020c), inhibited GbpB and GbpC gene expression in *S. mutans* during its biofilm formation (Kong et al., 2021). Curcumin also decreased GbpB gene expression in *S. mutans* biofilm by approximately 0.5 times (Li et al., 2020b).

Sortase A (SrtA) is a membrane enzyme that facilitates the anchoring of surface proteins to the cell wall (Mazmanian et al., 2000). SrtA is a virulence factor in oral gram-positive species, including *Streptococcus* spp., *Staphylococcus* spp., and *Enterococcus* spp. (Cascioferro et al., 2014). SrtA in *S. mutans* is essential for sucrose-independent adhesion, which facilitates the antigen I/II and Gbps attachment to the cell wall (Scharnow et al., 2019), while the srtA gene expression in *S. mutans* was downregulated by approximately one-fold after curcumin treatment (Li et al., 2020b). SrtA in *Enterococcus* spp. has played a key role in bacterial survival and is the potential treatment target to combat *Enterococcus spp.* (Cascioferro et al., 2014). Berberine, one of the main alkaloids isolated from *Rhizoma coptidis*, inhibited SrtA gene expression by 50% compared with the control group at 80 μg/ml (Chen et al., 2016). SrtA in *Staphylococcus* spp. also acts as a catalyst for the adhesion of proteins (such as FnBPA and FnBPB) to the cell wall (Paterson and Mitchell, 2004). Kaempferol, a typical flavonol, inhibited both SrtA activity and cell adhesion at 64 μg/ml, suggesting that its inhibition of biofilm formation was achieved by inhibiting SrtA activity to weaken the adhesion of *S. aureus* (Ming et al., 2017).

Als family, a class of cell wall glycoproteins, regulates cell adhesion and biofilm formation in *C. albicans* (Xu et al., 2022). Als1 and Als3 proteins play a vital role in adhesion to host endothelial and epithelial cells. Als5 is related to the binding to host extracellular matrix proteins (Ponde et al., 2021). Curcumin treatment downregulated the Als1 and Als3 gene expression, while upregulated Als5, indicating that curcumin reduced cell adhesion but enhanced cell aggregation (Alalwan et al., 2017). Garlic, a member of the *Liliaceae* family, also exerted inhibitory effects on Als1 and Als3 from *C. albicans* (Fahim et al., 2022).

Esp is a key surface protein of *Enterococci* that regulates the initial adhesion of cells to the surface. Berberine treatment deceased its gene expression in *E. faecalis* at 80 μg/ml, indicating the effectiveness of berberine in preventing *E. faecalis* cell adhesion (Chen et al., 2016).

##### Reduction in EPS generation

Extracellular polymeric substances is composed of proteins, polysaccharides, uronic acids, and nucleic acids, which significantly contribute to biofilm pathogenesis (Izadi et al., 2021). As a binding agent for initial bacterial adhesion, EPS also provides three-dimensional structure for oral biofilm. EPS can regulate the interactions among various bacterial species and protect the cells in the oral biofilm from antibiotics and environmental stresses (Lin et al., 2021). Thus, the inhibition of the EPS generation can be a promising target for the control of oral biofilm infection.

Extracellular polymeric substances of *S. mutans* is mainly produced by glucosyltransferases (Gtfs) and fucosyltransferases (Ftfs; Senadheera et al., 2005). Gtfs are a group of enzymes that split sucrose into glucose and fructose and then synthesize the EPS (Zhang et al., 2021b). Gtfs produce both insoluble and soluble glucans. GtfB produces insoluble glucans, GtfC can produce both insoluble and soluble glucans, while GtfD generates soluble glucans (Paes Leme et al., 2006; Wang et al., 2021). Insoluble glucans facilitate cell adhesion and provide a 3D structure in the biofilm, and soluble glucans act as an energy source and contribute to a low-pH microenvironment (Zhang et al., 2021b). Ftfs are encoded by the ftf gene, and they contribute to convert sucrose into extracellular fructose homopolymers (Burne and Penders, 1994).

Epigallocatechin gallate was able to inhibit EPS production as GtfBC gene expression of *S. mutans* were reduced by 77–90% at a sub-MIC concentration of 0.55 mg/ml (Schneider-Rayman et al., 2021). The Ftf gene expression of *S. mutans* was also downregulated by 70% after EGCG treatment for 24 h at this dosage (Schneider-Rayman et al., 2021). Sodium new houttuyfonate (SNH), a sodium bisulfite of houttuynia isolated from *Houttuynia cordata*, also showed an excellent ability to reduce EPS production. GtfBC of *S. mutans* was downregulated after the SNH treatment at 100 μg/ml (1/2 MIC), and GtfB expression was even downregulated by >50% (Shui et al., 2021). Curcumin treatment for 5 min was also downregulated the Ftf gene expression of *S. mutans* by 0.541-fold (Li et al., 2020b).

*Staphylococcus* spp. produce polysaccharide intercellular adhesin (PIA) to regulate their biofilm formation (Mack et al., 1994). PIA is encoded by a group of genes, including IcaADBC and the regulatory gene IcaR (Heilmann et al., 1996). Similar to EPS produced by *Streptococcus*, PIA is essential in the whole process of biofilm formation (Nguyen et al., 2020). In particular, PIA contributes to the hydrophobicity of *Staphylococcus epidermidis* and *S. aureus* cell surface and regulates the their initial adhesion during the biofilm formation (Nuryastuti and Krom, 2017). *Galla chinensis*, a natural product isolated from *Rhus chinensis*, suppressed IcaABCD gene expression in *S. aureus* at 7.81 μg/ml (Wu et al., 2019), while IcaABCD gene expressions in *S. epidermidis* was downregulated by 0.1–0.7-fold when treated with propolis (Ong et al., 2019), indicating their capabilities of anti-adhesion effects and furtherly inhibitory activities on the biofilm formation of *S. aureus* and *S. epidermidis*.

### Targeting at the biofilm formation

After the initial adhesion, the biofilm accesses the proliferation phase. In this phase, cells adhering to the surface continue to grow and produce EPS to form a biofilm matrix (Blackman et al., 2021). This structure provides a stable condition for microorganisms in the biofilm. The QS system represents the intercellular signaling in bacteria community, which regulates gene expression and actions in response to local cell density during the formation of the biofilm (Parsek and Greenberg, 2005).

#### Antibiofilm effect of natural products on biofilm formation

Natural products exerted a strong antibiofilm effects and reduced the total biomass of biofilm formation. *Punica granatum*, the pomegranate fruit, inhibited *S. mutans* biofilm formation by 94.76% at 1.56 mg/ml (Gulube and Patel, 2016). Biofilm formation of *S. aureus* was reduced by 45% after berberine treatment at 256 μg/ml (Guo et al., 2015), and coumarin can inhibit MRSA biofilm formation in a dose-dependent manner (0.25–4 μg/ml; Qu et al., 2020). Theaflavins, the major ingredients of tea polyphenols, reduced *P. gingivalis* biofilm formation by 50% at 1,000 μg/ml (Kong et al., 2015), while *thymoquinone*, the major component of black cumin essential oil, significantly inhibited *F. nucleatum* biofilm formation (Tada et al., 2020). The biofilm formation of *C. albicans* was completely inhibited by the treatment of 150 μM magnoflorine (an aporphine alkaloid; Kim et al., 2018).

#### Mechanisms inhibiting oral bacterial biofilm formation

##### Inhibition of cell proliferation and killing bacterial cells

Many natural products have microbiocidal properties on planktonic cells through various ways, including cell wall decomposition (Zhang et al., 2020b), cell membrane disruption (Abram et al., 2013), leakage of cell contents (Kang et al., 2015), inhibition of the synthesis of proteins and DNA (Fathima and Rao, 2016), and blockage of cell metabolism (Belenky et al., 2015). The bacterial cells on the surface of the biofilm can also be eradicated by natural products, then inhibit bacterial cell proliferation and biofilm formation. *Rhodiola rosea,* a medicine plant, reduced the viability of *S. mutans* by >99% (Zhang et al., 2020a). EGCG reduced the cell viability of *P. gingivalis* by 40% at 5 mg/ml, and the ratio of live cells were also significantly decreased after the exposure to EGCG (Asahi et al., 2014), which was result of the reduction in biofilm biomass.

##### Reduction in EPS Production

Extracellular polymeric substances, the major component of the biofilm, is not only pivotal in the adhesion process of cells to the surface but also important to the whole process of biofilm formation (Flemming and Wingender, 2010), thereby natural products which are capable to reduce the genes related to EPS generation (including Gtfs and Icas) can contribute to both inhibition of cell adhesion and biofilm formation. *Rhodiola rosea* reduced the total EPS *in S. mutans* biofilm at 0.25 μg/μL (Zhang et al., 2020a). *G. chinensis* inhibited EPS production of *S. aureus* biofilm by 44% at 7.81 μg/ml (Wu et al., 2019). The EPS inhibition of these natural products contributes to the total biofilm biomass reduction.

##### Inhibition of the QS system

The QS system regulates the bacterial behavior through small signaling molecules at the whole population levels, and this system is essential for biofilm formation in both gram-negative and gram-positive species (Abisado et al., 2018). The QS system can recognize the changes in the population density to regulate virulence factors (Holm and Vikström, 2014). The molecular mechanisms of the QS system are different in Gram-positive and Gram-negative species (Rutherford and Bassler, 2012; Papenfort and Bassler, 2016).

**Figure 2:**
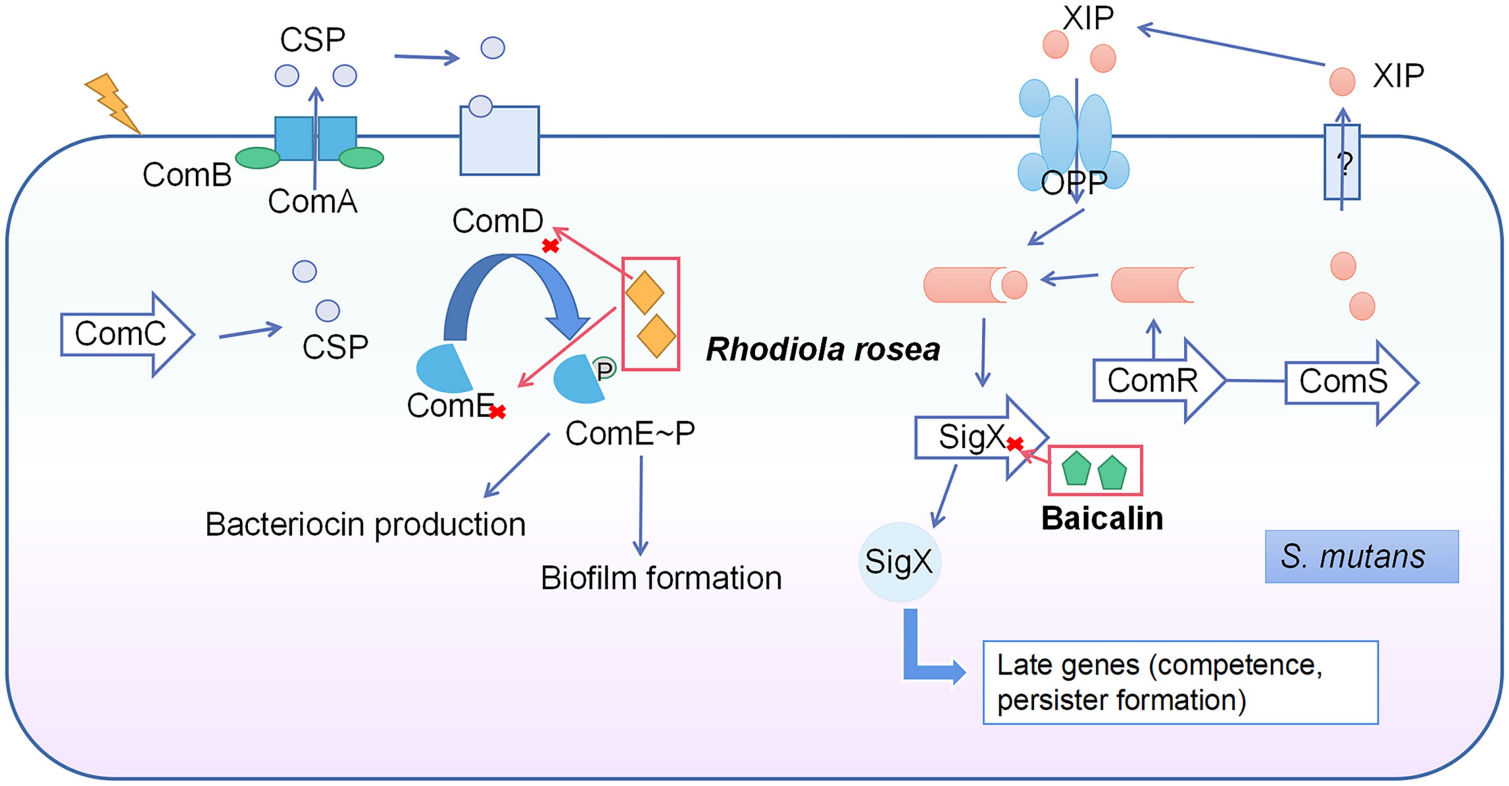
Quorum sensing system in *S. mutans* and the inhibitory effects of natural products on this system. The CSP-ComDE system is composed of a signal peptide (CSP, encoded by ComC) and the ComDE two-component system. During the cell density increase, the accumulated CSP interacts with ComD (membrane-bound histidine kinase receptor) directly to cause the phosphorylation and activation of ComE (the cytoplasmic response regulator). The activated ComE regulates the gene expression of bacteriocin production and biofilm formation. The ComRS system consists of signaling peptide pheromone (XIP, encoded by ComS) and a transcriptional regulator (ComR). The XIP interacts with and activates ComR to regulate the expression of ComX, and thus switches the genes related to competence and persister formation. *Rhodiola rosea* inhibited ComDE gene expression and baicalin inhibited ComX gene expression.

There are two main QS systems in the *S. mutans*: CSP-ComDE and ComRS systems (Kaur et al., 2015). The CSP-ComDE system is composed of a signal peptide (CSP, encoded by ComC; Leung et al., 2015) and the ComDE two-component system (Kaur et al., 2015; Figure 2). The ComRS system consists of the signaling peptide pheromone XIP (encoded by ComS) and a transcriptional regulator (ComR). The XIP interacts with and activates ComR to regulate the expression of ComX (Wenderska et al., 2017). *Rhodiola rosea* downregulated the ComDE gene expression at 0.25 mg/ml (Zhang et al., 2020a), while the gene expression of ComD was suppressed by >50% under SNH treatment at 100 μg/ml (Shui et al., 2021). Baicalin was able to inhibit ComX gene expression at 500 μg/ml (Elango et al., 2021).

**Figure 3:**
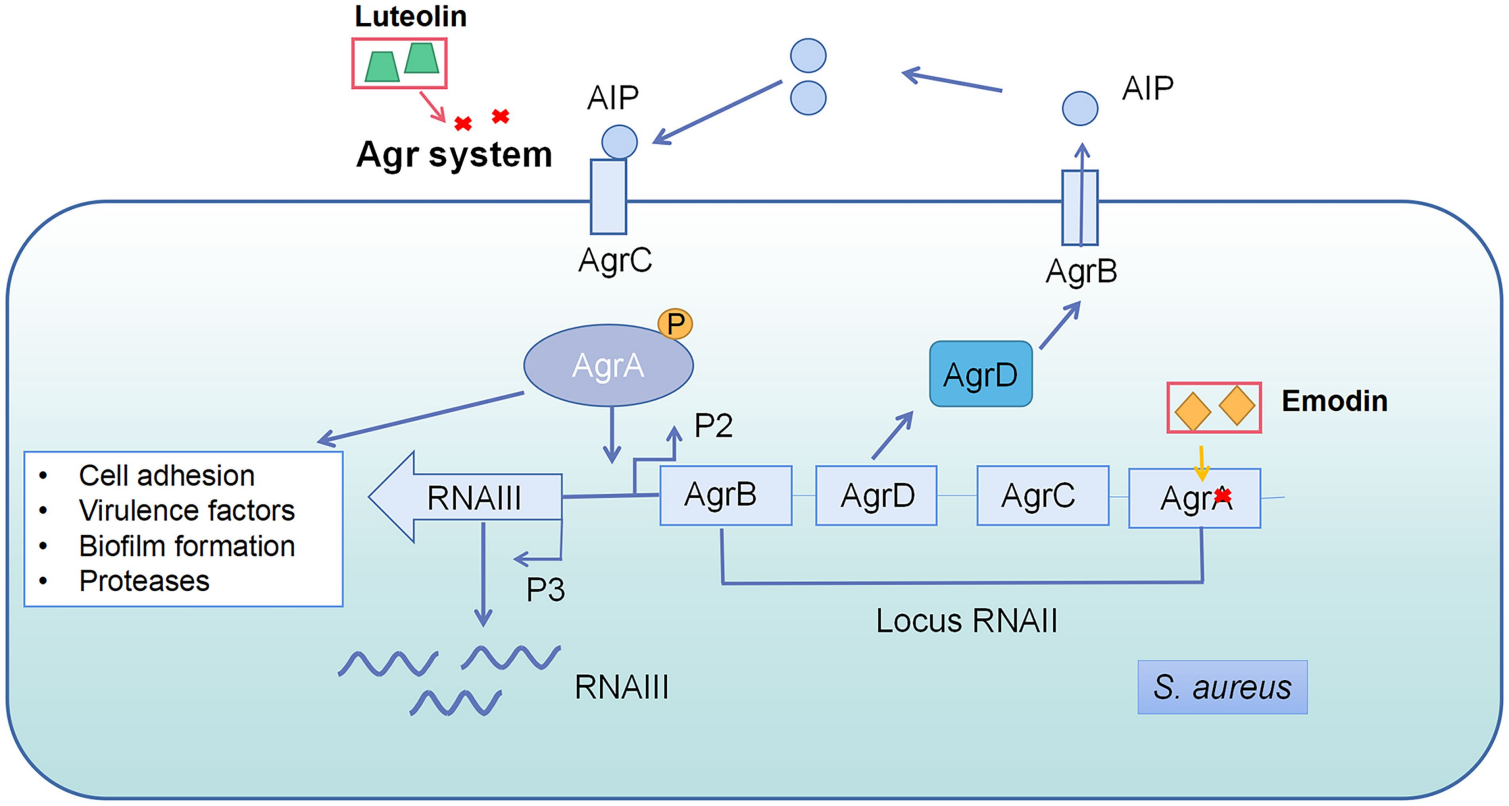
Quorum sensing system in *Staphylococcus aureus* and the interference of natural products on quorum sensing system. AgrD is the precursor of autoinducer peptides (AIP). AIP can be modified by AgrB and secreted into the matrix. AIP secreted by bacteria accumulates in the environment and binds to kinase receptors (AgrC) on the bacterial membrane to transmit signals, activating the related genes’ expression, such as RNAII and RNAIII. RNAIII regulates most QS-related genes, while some genes are controlled by AgrA directly. Emodin inhibited AgrA gene expression and luteolin interfered Agr system.

The accessory gene regulator (Agr) system is a key QS pathway in *S. aureus*, which is also common in gram-positive bacteria and essential for their virulence (Le and Otto, 2015). The Agr system contains four elements: AgrA, AgrB, AgrC, and AgrD (Figure 3; Schilcher and Horswill, 2020). Emodin reduced the expression of AgrA gene by 2.2 folds at 4 μg/ml (Yan et al., 2017). Luteolin, a bioactive component in fruits and vegetables, decreased the pathogenesis of *S. aureus* through interference of the Agr system. The wild strain exhibited weaker virulence compared with △*AgrBCD*, including biofilm formation, initial adhesion, and virulence gene expression, suggesting that the Agr system is the target of luteolin against *S. aureus* (Yuan et al., 2022).

**Figure 4:**
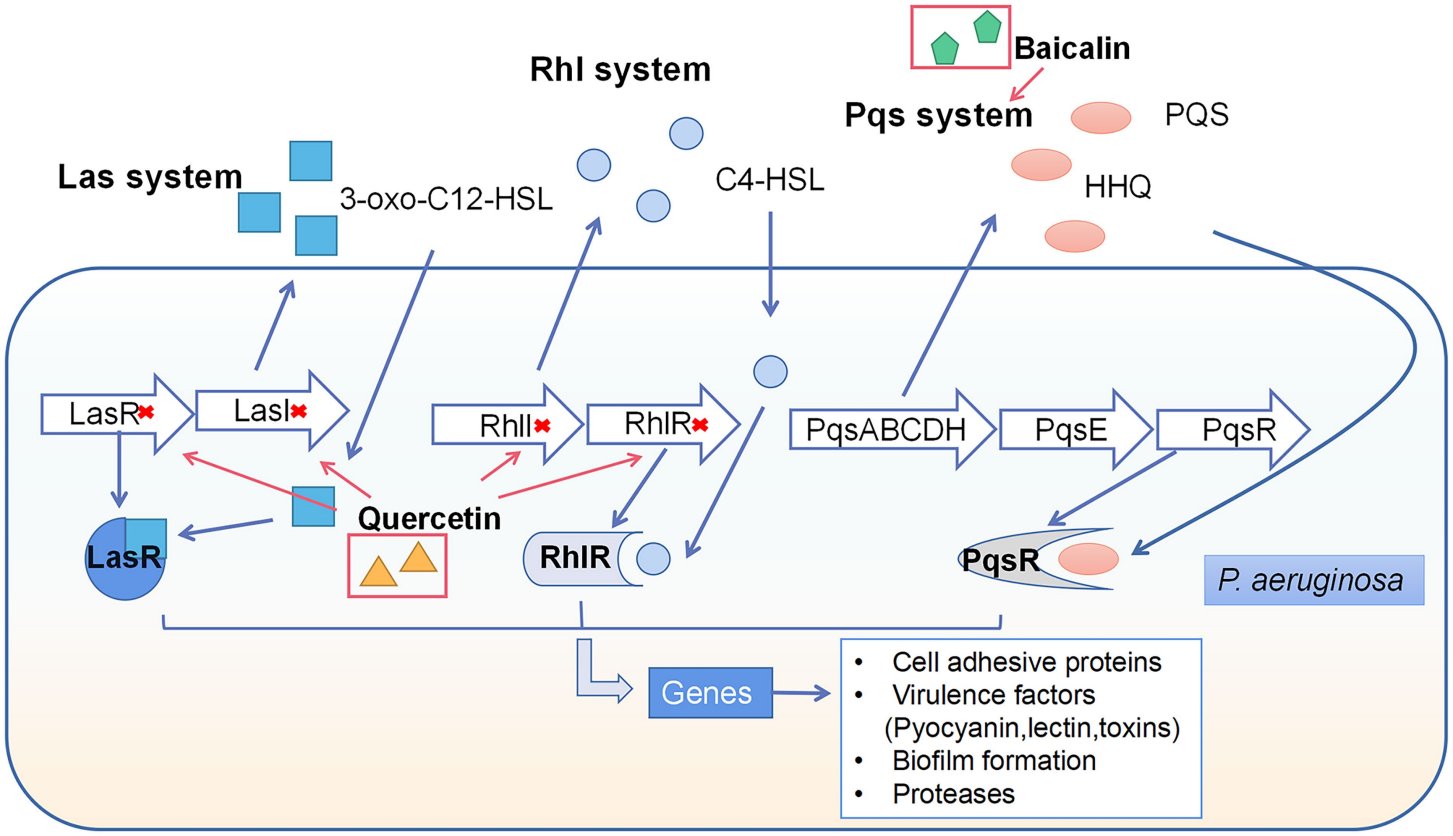
Quorum sensing system in *P. aeruginosa* and the interference of natural products on quorum sensing system. There are three key pathways in the *P. aeruginosa* QS system: two LuxI/LuxR-type QS pathways and the pseudomonas quinolone signal (PQS) system, named Las, rhl, and pqs. The synthesis of AI’s 3-oxo-C12-HSL and C4-HSL is modulated by Las and Rhl, which serve as their autoinducers, respectively. In addition, alkyl-4-quinolones (AQs), including PQS and HHQ, are signal molecules in the PQS pathway. Interconnections between the three pathways regulate the activity of the QS system, resulting in changes in cell adhesive proteins, virulence factors, biofilm formation and proteases. Quercetin inhibited LasI, LasR, RhlI, RhlR gene expression and baicalin inhibited Pqs system.

In gram-negative bacteria, such as *P. aeruginosa*, the autoinducer acyl-homoserine lactones (AHL) acted as QS molecule can bind to cytoplasmic receptors to regulate bacterial actions (Galloway et al., 2011). There are three key pathways in *the P. aeruginosa* QS system: two LuxI/LuxR-type QS pathways (Rutherford and Bassler, 2012) and the pseudomonas quinolone signal (PQS) system, named Las, Rhl, and Pqs (Guzzo et al., 2020; Figure 4). Quercetin (QCT), a flavonol extracted from vegetables and fruits, significantly suppressed the expression of LasI, LasR, RhlI, and RhlR, which were related to Las and Rhl pathways (Ouyang et al., 2016). Additionally, baicalin inhibited the Type III secretion system (a virulence factor for infection) through inhibiting the PQS system (Zhang et al., 2021a).

#### Mechanisms of inhibiting fungal biofilm formation

*Candida albicans* is a major opportunistic fungal pathogen in oral cavity and highly associated with several oral diseases, such as caries (especially root caries; Xiong et al., 2020; Du et al., 2021) and oral candidiasis (Zhou et al., 2021). Berberine induced a decrease in the viability of *C. albicans* biofilms with the actions on the integrity of plasma, mitochondrial membranes, and DNA (Da Silva et al., 2016). The viability rate of *C. albicans* after the treatment with berberine was reduced by 43.54% (Xie et al., 2020).

The hyphal form is an important phase of *C. albicans* biofilm formation and is the key virulence factor (Chen et al., 2020). Berberine inhibited the yeast to hyphae growth of *C. albicans* and significantly downregulated hypha growth-related gene expression (Efg1, Hwp1, Ece1, and Als1) at the sub-MIC concentration (8–128 μg/ml; Huang et al., 2020b). After SNH treatment, the transcriptome sequencing showed that the biofilm formation-related genes in the Ras1-cAMP-Efg1 pathway (Als1, Ala1, Als3, Eap1, Ras1, Efg1, Hwp1, and Tec1) were downregulated (Wu et al., 2020). The combination of garlic and bakuchiol significantly reduced Als3 and Sap5 gene expressions associated with hyphal growth (Fahim et al., 2022).

Farnesol and tyrosol are the major QS signaling molecules found in *C. albicans* (Davis-Hanna et al., 2008). Farnesol is an autoregulatory molecule that inhibits the yeast phase’s transformation to the hypha phase (Xu et al., 2022). Farnesol is also widely distributed in propolis and fruits (Costa et al., 2021). Farnesol inhibited the hyphal growth by repressing Ras1-Cdc35-PKA-Efg1 pathway, indicating that farnesol is a promising molecule in inhibiting biofilm formation of *C. albicans* by interfering with the QS system (Davis-Hanna et al., 2008).

### Eradication of mature biofilms

The EPS acts as a protective multifunctional scaffold in the mature biofilm (Flemming and Wingender, 2010). The cells in the biofilm are closely aggregated and facilitates interactions and food chains among proximal neighbors (Kuramitsu et al., 2007). In mature stage, biofilm shows an increased tolerance to antimicrobial agents (Cadena et al., 2019). For example, the minimum inhibitory concentration (MIC) of CHX to kill *Streptococcus sobrinus* in the established biofilm increased 300 times compared with planktonic cells (Shani et al., 2000). Therefore, many refractory infectious biofilm-related diseases caused by mature biofilms are difficult to remove (Noiri et al., 2002).

Natural products have the potential to remove mature microbial biofilms. Propolis was effective in eradicating *Candida.* spp. biofilms, which could eradicate 50% *Candida*. spp. biofilm with the concentration of 2.5% (Gucwa et al., 2018). Propolis also reduced the viability of *S. aureus* biofilm by 92.9% at a concentration of 125 μg/ml but did not decrease the total biomass. The results demonstrated that propolis penetrated the biofilm and killed the cells inside but did not decrease the total biomass of mature biofilms (De Oliveira Dembogurski et al., 2018). In another study, the treatment with propolis reduced *S. aureus* biofilm biomass by >50% at 200 μg/ml, and the thickness of biofilm decreased by 47–87% in different isolates (Bouchelaghem et al., 2022).

### Inhibition of biofilm dispersion

When the nutrients are limited and waste products in the biofilm accumulate a lot, biofilm dispersion allows microorganisms to depart from biofilms and to colonize new niches (Solano et al., 2014). The biofilm dispersion made infection worse and hard to control, and even caused an acute infection, such as sepsis (Lister and Horswill, 2014). Sodium houttuyfonate was able to inhibit *P. aeruginosa* biofilm dispersion through the inhibition of chemotaxis transducer protein BdlA gene expression (a key gene that regulates the dispersion response of *P. aeruginosa*; Wang et al., 2019). Phenol-soluble modulins (PSMs) are biofilm-dispersion-associated factors related to *S. aureus* infection (Zheng et al., 2018). Berberine inhibited PSMs production as evidenced by the calculation of amyloid fibril formation (Chu et al., 2016). Reducing biofilm dispersion is of great significance in controlling infection spread in clinical practice (Rumbaugh and Sauer, 2020).

### Combinational application of natural products and other strategies

#### Combination of natural products and nanoparticles

Nanoparticles have a significant potential in the delivery of drugs against oral biofilm due to their flexible properties (Benoit et al., 2019). Some natural products extracted by oil and ethanol, such as propolis and curcumin, have poor water solubility, which limits their clinical usage (Kubiliene et al., 2015). Nanoparticles can be designed to enhance drug solubility. Propolis-loaded poly (lactic-co-glycolic acid, PLGA) nanoparticles were synthesized to enhance the solubility of propolis, and this nanoparticles showed excellent antibiofilm effects on *C. albicans* (Iadnut et al., 2019). A combination of nanoparticles and natural products may result in synergistic antibiofilm effects due to the high surface area-to-volume ratios of nanoparticles (Shrestha and Kishen, 2016). Pterostilbene, a kind of *Vitis*-inducible phytoalexins, showed a much higher antibiofilm effect after being loaded in PLGA nanoparticles (Simonetti et al., 2019). Furthermore, the combination with nanoparticles extended the release time of natural products, which is important for long-term antibiofilm effects (Maghsoudi et al., 2017). For example, Berberine in nanoparticles exerted a better *S. aureus* biofilm removal ability than berberine alone, which might be attributed to the spontaneous adhesion property and continuous release characteristics of nanoparticles (Huang et al., 2020a). The integration of nanoparticles and natural products enhanced the efficacy of natural products for multifunction properties at the same time, providing a great potential in clinical applications (Yu et al., 2017, 2021).

#### Combination of natural products and antibiotics

The combination of products and antibiotics is a practical way to reduce the development of antibiotic resistance and also to reduce the toxicities or side effects of some antibiotics by decrease the antibiotic dosages against biofilms (Li et al., 2013; Zhu et al., 2021). Artemisinin, a famous antimalarial sesquiterpene lactone extracted from the traditional Chinese herb *Artemisia annua* L, was able to increase the cell membrane ergosterol levels of *C. albicans* to synergize with amphotericin B to inhibit *C. albicans* and oral candidiasis (Zhu et al., 2021). The combination of berberine and fluconazole showed synergic effects on *C. albicans* biofilms by enhancing the susceptibility of *C. albicans* to fluconazole. Interestingly, the antibiofilm effect was related to berberine in a concentration-dependent manner instead of fluconazole, indicating that berberine played a major role in the antifungal effect (Li et al., 2013). Similarly, natural products in combination can improve the antibiofilm properties in the elimination of mature biofilm. The combination of berberine and fusidic acid significantly inhibited cell viability in *S. aureus* mature biofilms, while the single drugs did not show any antibiofilm effects (Liang et al., 2014).

#### Combination of natural products and photodynamic therapy

Photodynamic therapy (PDT) is an effective method in cancer management (Li et al., 2020a), periodontitis (Manresa et al., 2018), and oral mucosal diseases (Cosgarea et al., 2020). In recent years, it has been demonstrated that PDT was able to enhance the activities of natural products, even on drug-resistant microorganisms. Many natural products have shown stronger antibiofilm effects in combination with PDT, such as emodin (Pourhajibagher et al., 2022), propolis (Afrasiabi et al., 2020), and curcumin (Santezi et al., 2018). The combination of curcumin and PDT is effective in infection control (Polat and Kang, 2021). Combination of curcumin and PDT reduced *P. aeruginosa* biofilm formation through interfering quorum sensing network, and importantly, the combination significantly enhanced antibiofilm effect compared with curcumin alone (Abdulrahman et al., 2020). The combination of natural products and PDT provides a new direction in managing biofilm-related oral infections.

#### Combination of two natural products

Propolis and carnosic acid (a compound extracted from rosemary) showed synergistic effects against *C. albicans*, while the 1:4 ratio of carnosic acid and propolis resulted in a best decrease in *C. albicans* survival rate and biofilm formation (Argüelles et al., 2020). Curcumin and berberine co-encapsulated in liposomes showed synergistic effects against MRSA by reducing their MICs by 87 and 96% compared with single drugs and the biofilm formation also significantly decreased (Bhatia et al., 2021). These results indicate that the combination of different natural products with different antimicrobial mechanisms can enhance their activities even on drug-resistant pathogens.

## Effects of natural products on multi-species biofilms

Multi-species biofilms represent the most important lifestyle of oral microbes in oral cavity (Marsh et al., 2011; Yang et al., 2011). The interactions among microbes regulate the structure and function of biofilms and significantly influence the biofilm formation (Yang et al., 2011; Deng et al., 2019a,b). Microorganisms in the multi-species biofilm enhance the have metabolism efficient, tolerance to inhibitory agents and virulence (Marsh et al., 2011).

Natural products have shown effective activities in multi-species biofilms. Curcumin reduced the biomass and viability of *C. albicans* and *S. mutans* dual-species and mono-species biofilms. Interestingly, more eradication of *S. mutans* was found indicating that the effect of curcumin on *S. mutans* was enhanced in the *C. albicans* and *S. mutans* dual-species biofilm. Moreover, curcumin also blocked the EPS generation of *C. albicans* and *S. mutans* dual-species biofilm through the inhibitions on the QS system, EPS generation, and Als protein production (Li et al., 2019). Brazilian red propolis (BRP) showed an antibiofilm effect on multi-species biofilm composed of periodontopathogens (34 species). The metabolism of multi-species biofilms decreased to 45 and 55% after 800 μg/ml BRP and 0.12% CHX treatment, respectively. Biofilm cells were reduced to 10 and 5% after being treated with 1,600-μg/mL BRP and 0.12% CHX, respectively. In addition, BRP had a significant antibiofilm effect on species in the orange-complex group, while 0.12% CHX did not have such an effect (Miranda et al., 2019). BRP extract (400 μg/ml) also exhibited an almost equal ability to that of amoxicillin (54 μg/ml) to remove red-complex of multi-species subgingival mature biofilms (De Figueiredo et al., 2020). These findings indicated its promising effects in periodontitis treatment.

In recent years, the microcosm biofilm model (consisting of natural oral microbiota) has been established to better simulate the *in vivo* oral cavity conditions (Garcia et al., 2021). *G. chinensis* extract inhibited biofilm formation in both nascent and mature microcosm biofilms, and the acid metabolism in biofilms was also inhibited (Cheng et al., 2011). C*offea canephora* reduced mixed biofilms formed from the pooled human saliva by 15.2% at the concentration of 20% (Antonio et al., 2012). *Psidium cattleianum* leaf extract reduced *in situ* oral biofilms formation and EPS production at 167 mg/ml for 1 min per 12 h for 14 days treatment (Brighenti et al., 2012). Natural products contained in clinical products also showed antibiofilm effects on microcosm biofilms. *Myrcia bella* Cambess. and *Matricaria chamomilla* L. in the toothpastes reduced the bacterial number in the microcosm biofilms. The enamel demineralization assay revealed that *Vochysia tucanorum*, *Myrcia bella*, *Matricaria chamomilla*, and Myrrha & propolis toothpastes reduced mineral loss and lesion depth compared with the placebo group (Braga et al., 2022). Another study demonstrated that natural products (including Commiphora myrrha resin extract, propolis extract) in the commercial toothpastes reduced microcosm biofilm viability and increased mineral loss (Braga et al., 2019). These results indicated the clinical potential in the control of oral biofilms.

## Natural products as an alternative treatment for oral infection

The strong antibiofilm effects of natural products on both mono-species biofilms and multi-species biofilms with various mechanisms highlight their clinical oral disease controls. Several *ex vivo* & *in vivo* studies and clinical trials have been implemented to evaluate the efficacity of natural products, especially in caries and periodontitis.

In dental caries, many natural products, such as propolis and sodium new houttuyfonate, were able to inhibit *S. mutans* biofilm virulence, EPS production and QS system (Veloz et al., 2016; Shui et al., 2021). Propolis inhibited *S. mutans* biofilm formation and dental caries development in a rat model (Duarte et al., 2006), while magnolol and honokiol extracted from magnolia bark reduced the biofilm formation by an *in vivo* Germ-kill model (Greenberg et al., 2007). Importantly, a randomized controlled trial also showed that magnolia bark have encouraging results in maintaining oral health by reducing *S. mutans* proliferation, plaque acidogenicity and bleeding on probing (Campus et al., 2011).

In periodontitis, the natural products EGCG showed a great antibiofilm effect on *P. gingivalis* (Fournier-Larente et al., 2016) and *F. nucleatum* (Ben Lagha et al., 2017). BRP extract (400 μg/ml) used in multi-species biofilms exhibited an almost equal ability to that of amoxicillin to eliminate the red-complex of multi-species subgingival mature biofilms which suggested its promising action in periodontitis treatment (De Figueiredo et al., 2020). The mouth rinses containing *Aloe vera* reduced the plaque and gingival inflammation and finally significantly reduced clinical scores indicating the potential application of *Aloe vera* in periodontitis (Laleman and Teughels, 2020).

Natural products have strong antibiofilm effects on key pathogens of other oral infectious diseases, and the diversity, efficiency and safety of natural products make them the alternative agents to antibiotics against biofilms even from the drug-resistant strains.

## Prospects of maintaining oral microecology balance

The resident microflora in the oral cavity of healthy individuals has great significance in maintaining health, preventing foreign pathogens colonization and contributing to host physiology (Rosier et al., 2018). The oral microbiota in a healthy condition is more stable than other microbial communities (Zhou et al., 2013) and resists diseases (Rosier et al., 2018). However, many factors can disturb the balance, including systematic diseases, unhealthy diet, and poor oral hygiene, and the usage of broad spectrum antibiotics (Lamont et al., 2018). In caries, an unhealthy diet (fermentable carbohydrates in high amounts and frequency) often results in the accumulation of fermentation extract (organic acid; Lamont et al., 2018). When the acid caused the decrease in pH subsequently, the oral microbiota shifted toward the adaption of the low pH conditions and became the cariogenic microbiota (Pitts et al., 2017). It is important to restore the microecological balance instead of only killing the oral microbes indiscriminatingly (Baker et al., 2017).

BRP used in a periodontitis model showed an excellent antibiofilm effect on the red-complex and orange-complex species, but showed less effect on other species, indicating that this compound was less harmful to beneficial microorganisms (Miranda et al., 2019; De Figueiredo et al., 2020). Currently, the influence of natural products on the whole oral microflora and how does the microflora change after drug treatment remain unclear; however, natural products have shown potential to restore microecological balance compared with broad-spectrum antibiotics (Melander et al., 2020).

## Discussion and future prospective

Biofilms represent the common form of microorganisms in the oral cavity and the dysbiosis of biofilms are highly related to many oral infectious diseases, such as caries and periodontitis (Marsh and Zaura, 2017). Natural products from traditional medicine are promising agents against oral biofilms due to their excellent antibiofilm effects, relatively low cost, and safety (Atanasov et al., 2021). However, there are still many concerns on natural product applications in clinical practice. For example, many of them have low solubility, which greatly limits their usage. Meanwhile, the active ingredients of herbal medicines are complex, and currently, microbiocidal and antibiofilm mechanisms of most herbal medicines have not been fully elucidated. Although many studies performed clinical trials using many active ingredients such as tea, propolis, and *Aloe vera*, showing encouraging results (Laleman and Teughels, 2020), the toxicity to normal cells of natural products is another concern and need to be further explored. Previous study revealed the nephrotoxicity of traditional medicine (including anthraquinones and flavonoids; Yang et al., 2018), suggesting the necessity of a safety evaluation before the use of natural products in clinical practice.

Natural products have a broad prospective in oral clinical application. However, the effects of natural products on the local microbiota and their impact on the local microecological balance after drug treatment should be further explored. More clinical trials and safety test of natural products are also needed, while our review summarized and discussed the potential effects of natural products from traditional medicine against oral biofilms to highlight the importance of further investigations on natural products in treating oral infectious diseases.

## Author contributions

YC wrote the original draft of the manuscript, compiled the tables, and made the figures. YW, MJ, YL, HZ, and YY collected the data. LZ and BR revised the manuscript. All authors contributed to the article and approved the submitted version.

## Funding

This work was supported by grants from the National Natural Science Foundation of China (grant nos. 82071111, 81870778, 81991500, 81600858, and 81991501), the Project of the Science and Technology Department of Sichuan Province (grant nos. 2020YFSY0019 and 2021YFQ0064), and Applied Basic Research Programs of Sichuan Province (2020YJ0227).

## Conflict of interest

The authors declare that the research was conducted in the absence of any commercial or financial relationships that could be construed as a potential conflict of interest.

## Publisher’s note

All claims expressed in this article are solely those of the authors and do not necessarily represent those of their affiliated organizations, or those of the publisher, the editors and the reviewers. Any product that may be evaluated in this article, or claim that may be made by its manufacturer, is not guaranteed or endorsed by the publisher.
